# A phase 1b/2 study of cabozantinib in combination with pembrolizumab in advanced cutaneous melanoma

**DOI:** 10.1002/cncr.70326

**Published:** 2026-02-17

**Authors:** Yousef Zakharia, Donghyun Kim, Michele Freesmeier, Melanie Frees, Sarah Mott, Varun Monga, Douglas Laux, Asad Javed, John Rieth, John Smestad, Mohammed Milhem

**Affiliations:** ^1^ Division of Hematology, Oncology, and Blood and Marrow Transplantation Department of Internal Medicine University of Iowa Health Care Iowa City Iowa USA; ^2^ Mayo Clinic Comprehensive Cancer Center Phoenix Arizona USA; ^3^ University of Iowa Holden Comprehensive Cancer Center Iowa City Iowa USA; ^4^ Division of Hematology and Oncology, Department of Medicine University of California San Francisco San Francisco California USA; ^5^ Physician‐Scientist Training Program University of Iowa Hospitals and Clinics Iowa City Iowa USA

**Keywords:** combination therapy, immune checkpoint inhibitor, skin cancer

## Abstract

**Background:**

Pembrolizumab is approved for advanced cutaneous melanoma (aCM). Cabozantinib, an oral multi‐tyrosine kinase inhibitor, has demonstrated antitumor activity as monotherapy or in combination with anti‐PD‐1 therapy in malignancies. The objective of this study was to determine the safety and efficacy of cabozantinib and pembrolizumab for patients with aCM.

**Methods:**

This phase 1b/2 study enrolled 28 patients with unresectable aCM. In phase 1b, escalating dose levels of cabozantinib (20 mg, 40 mg, and 60 mg) were administered concurrently with pembrolizumab 200 mg intravenously every 3 weeks using a 3 + 3 design to determine recommended phase 2 dose (RP2D). In phase 2, patients received cabozantinib at RP2D in combination with pembrolizumab using a Simon 2 stage design. Primary end point of phase 2 was overall response rate (ORR). Secondary end points included disease control rate, progression‐free survival (PFS), and overall survival (OS).

**Results:**

Phase 1b enrolled eight patients, cabozantinib 40 mg daily dose level was selected as RP2D. Response rate in stage 1 exceeded predefined criteria for proceeding to expansion phase; however, phase 2 was terminated after 20 patients due to low accrual in the setting of evolving standard of care. Among all treated patients, the most common grade ≥3 toxicities were hypertension (*n* = 10, 36%), hypokalemia (*n* = 5, 18%), hypophosphatemia (*n* = 4, 14%), and ALT elevation (*n* = 4, 14%). Among 20 patients treated in phase 2, ORR was 45%, median PFS was 6.6 months, and median OS was 29.5 months.

**Conclusions:**

Cabozantinib and pembrolizumab combination was explored in treatment‐naive aCM. Toxicity was consistent with known profiles, but discontinuation rates were notable.

## Abstract 2

This phase 1b/2 study explored the safety and preliminary efficacy of the cabozantinib 40 mg daily and standard dose pembrolizumab combination therapy in frontline setting for patients with advanced or metastatic cutaneous melanoma. Toxicity was consistent with known safety profiles and efficacy data suggested an overall response rate of 45%, median progression‐free survival of 6.6 months, and median overall survival of 29.5 months.

## INTRODUCTION

Before the advent of immune checkpoint inhibitor (ICI) therapy, metastatic melanoma was a notoriously grim disease, with few patients surviving longer than 5 years after diagnosis.^1^ Treatment options generally included cytotoxic chemotherapies such as dacarbazine, temozolomide, or platinum agents, with overall response rates (ORR) of 10%–20% and 5‐year overall survival (OS) under 10%.^2^, ^3^, ^4^, ^5^ Real‐world retrospective data from the IMAGE study reported median progression‐free survival (PFS) of 2.6 months and median OS of 8.8 months on these therapies.^6^ High dose interleukin‐2 (IL‐2) was the first immunotherapy approved by the Food and Drug Administration (FDA) for melanoma in 1992, with ORR of 16%, including complete response (CR) rate of 6%, but with associated severe risk of treatment‐related toxicities including 2% risk of death.^7^ For patients who did not achieve CR, treatment responses were generally short‐lived, with median PFS of 5.9 months after achieving partial response (PR).

Ipilimumab, a cytotoxic T‐lymphocyte–associated antigen 4 (CTLA‐4) antibody, was the first FDA‐approved immune checkpoint inhibitor therapy in 2011 based on the phase 3 trial data demonstrating ORR 10.9% and median OS of 10.1 months in patients with metastatic melanoma.^8^ Anti‐programmed cell death‐1 (PD‐1) ICIs including pembrolizumab and nivolumab, first approved for advanced melanoma in 2014, further improved outcomes with ORR in trials reported at 33%–45% (31%–33% based on real‐world data),^9^, ^10^ median PFS of 8.4 for pembrolizumab and 5.1 months for nivolumab, and median OS of 32.7 months for pembrolizumab and 37.5 months for nivolumab per data from KEYNOTE‐006 and CheckMate 066 trials, respectively.^11^, ^12^ Combination ICI regimens subsequently emerged with further enhanced efficacy. The pivotal CheckMate 067 trial demonstrated that the combination of ipilimumab with nivolumab resulted in ORR of 58% with PFS 11.5 months, and median OS 71.9 months, resulting in FDA approval in 2015.^13^ A second combination regimen of nivolumab with anti‐lymphocyte–activation gene‐3 agent relatlimab was subsequently approved in 2022 based on data from the RELATIVITY‐047 trial showing ORR 43.9%, median PFS 10.2 months, and median OS 51.0 months, with lower risk of severe adverse events compared to ipilimumab with nivolumab.^14^

In addition to immunotherapy options, for patients with *BRAF c.1799T>A* (*BRAF V600E*) mutation, BRAF/MEK tyrosine kinase inhibitor (TKI) combinations have been approved options since 2014, although ICI remains preferred in frontline setting.^15^ Since 2015, oncolytic virus (OV) therapy with talimogene laherparepvec has also been FDA‐approved for advanced melanoma patients with injectable cutaneous, subcutaneous, or lymph node lesions based on data from the phase 3 OPTiM trial demonstrating ORR 31.5%, durable response rate 19%,^16^ although it is generally not viewed as effective in addressing visceral or intracranial disease. For patients with prior progression on ICI, tumor‐infiltrating lymphocyte therapy with lifileucel has been approved since 2024 based on phase 2 data demonstrating ORR 36%, although with 7.5% risk of treatment‐related mortality.^17^

Despite these advances in therapy, clinical outcomes for patients with advanced melanoma remain constrained by generally few treatment options, low response rates, development of resistance to immunotherapy, and tolerability of treatment. Multiple strategies have been proposed to augment immunotherapy efficacy in melanoma including individualized cancer vaccines, in situ vaccines, OVs, cytokines, Toll‐like receptor (TLR) agonists, adoptive cell therapy, receptor tyrosine kinase inhibitors (TKI), metabolic modulation, epigenetic modulation, low dose chemotherapy, and radiation.^18^ In the present work, we further explore the application of a TKI strategy to augment pembrolizumab systemic antitumor immunity.

Multitarget TKIs blocking vascular endothelial growth factor receptor (VEGFR) 1‐3, which suppress angiogenesis and block hypoxic/pseudohypoxic signaling within tumors, have been increasingly recognized as therapeutic immuno‐modulators that can potentiate cancer vaccines,^19^ anti‐PD1 activities,^20^ and immunogenic cell death^21^ making multi‐TKIs promising agents to be used in combination with ICI to treat cancers.^22^ Such combinations have therefore been tested in multiple phase 3 trials across a variety of solid cancer types^23^ and have become standard for advanced renal cell carcinoma (RCC)^24^, ^25^, ^26^, ^27^ and in endometrial cancer following progression on platinum doublet chemotherapy.^28^

Here, we present the results of a phase 1b/2 clinical trial of cabozantinib in combination with pembrolizumab in patients with previously untreated metastatic or recurrent unresectable cutaneous melanoma. Cabozantinib is an oral multi‐TKI, of which its primary targets include MET, VEGFR2, RET, and AXL.^29^ Among the multiple possible TKI combinations, we selected cabozantinib for use in this study based on prior randomized phase 2 data in pre‐ICI era suggesting single agent antitumor efficacy of cabozantinib in patients with melanoma.^30^ Given these prior clinical data showing single‐agent activity, along with the growing body of evidence suggesting a basis for robust synergy of cabozantinib and other TKIs in potentiating antitumor immunity with given concurrently with anti–PD‐1 ICI,^20^, ^21^, ^22^, ^24^, ^29^, ^31^ we hypothesized that concurrent use of cabozantinib would enhance the antitumor activity of the standard of care pembrolizumab in advanced melanoma without significant increase in treatment‐limiting toxicities in the front‐line setting. We reasoned that such an ICI and TKI combination would potentially meet an unmet clinical need for patients with advanced melanoma who are unfit for dual ICI therapy.

Since the time of conception of this study, the field of melanoma therapy has evolved considerably, ultimately leading to the premature termination of this study. Specifically, descriptive data from CheckMate 511 trial published in 2019 comparing ipilimumab 1 mg/kg and nivolumab 3 mg/kg with ipilimumab 3 mg/kg and nivolumab 1 mg/kg in advanced melanoma showed numerically similar oncologic outcomes but with substantially reduced risk of grade ≥3 treatment‐related adverse events (TRAEs, 34% vs. 48%, descriptive *p* value = .006).^32^ A second major advance to the frontline management of melanoma was the FDA approval of nivolumab/relatlimab in 2024, which is a tolerable regimen with oncologic outcomes superior to anti–PD‐1 monotherapy.^14^ These two clinical advances in frontline therapy for melanoma effectively addressed the therapy gap that was the subject of this study, although the approach being investigated remains highly relevant, as strategies to augment ICI efficacy remain a subject of active investigation, especially in the relapsed and refractory setting.^18^, ^31^, ^33^

## MATERIALS AND METHODS

### Study design

This was a single‐center, open‐label, phase 1b/2 study designed to evaluate safety and tolerability, establish the recommended phase 2 dose (RP2D), and estimate the preliminary antitumor activity of cabozantinib in combination with pembrolizumab for adult patients with unresectable in‐transit (stage IIIC) or metastatic (stage IV) cutaneous melanoma (NCT03957551).

The primary objective of phase 1b was to establish the RP2D of cabozantinib using a 3 + 3 design with a fixed dose of pembrolizumab (200 mg intravenously every 3 weeks) and three dose levels of cabozantinib (20, 40, and 60 mg) administered orally daily. The daily 40 mg dose was previously established as the recommended starting dose for the combination treatment with nivolumab in advanced genitourinary cancers.^24^ DLTs were defined as adverse events deemed related to the study therapy that occurred during the 21 days from the first dose of cabozantinib, including asymptomatic grade 4 neutropenia lasting for >5 days, febrile neutropenia (absolute neutrophil count <1000/mm^3^ with a temperature of ≥38.3ºC), grade ≥3 neutropenic infection, grade ≥3 thrombocytopenia with bleeding, asymptomatic grade 4 thrombocytopenia lasting for >5 days, or any non‐hematologic grade ≥3 adverse events at least possibly related to study treatment of any duration. Transient (≤72 hours) abnormal laboratory value without associated clinically significant signs or symptoms, nausea, vomiting, or diarrhea adequately controlled with optimal supportive care, hypertension, or grade 3 fatigue in a patient with baseline grade 1 or 2 fatigue was not considered DLTs. Toxicity was graded according to the National Cancer Institute Common Terminology Criteria for Adverse Events, version 4.03.

The primary objective of phase 2 was to evaluate the preliminary efficacy of the established dose of cabozantinib in combination with pembrolizumab as measured by best ORR (CR + PR) with the combination of agents in patients with unresectable stage III or stage IV melanoma. The primary end point for phase 2 was ORR, with secondary end points including disease control rate (DCR), PFS, and OS.

In addition to a baseline computed tomography (CT) scan of the chest, abdomen, and pelvis obtained within the 28 days before day 1 of treatment, subsequent CT scans were obtained every 12 weeks, with a final CT scan obtained either at the end‐of‐treatment visit at 30 days (±7 days) from the date of last dose of study drug or before another treatment starts, whichever occurs first. Patients were evaluated for survival and progression post treatment every 3 months for 2 years, every 6 months for 3 years, and yearly until death.

### Patients

Eligible participants were ≥18 years or older with histologically or cytologically confirmed unresectable in‐transit (stage IIIc) or metastatic (stage IV) cutaneous melanoma, who have not received treatment for advanced melanoma. Prior therapy could include anti–PD‐1, anti–PD‐L1, or anti–PD‐L2 therapy in the adjuvant setting ≥6 months before developing metastatic relapses, or BRAF and/or MEK inhibitor for advanced melanoma. Other key inclusion criteria included measurable disease by Response Evaluation Criteria in Solid Tumors v1.1; adequate bone marrow, liver, and renal functions, and Eastern Cooperative Oncology Group performance status of 0 to 2. Key exclusion criteria included prior exposure to cabozantinib for any indication, prior exposure to any small molecule TKI within 2 weeks before the first dose of study treatment, autoimmune disease requiring immunosuppressive agents, ocular or mucosal melanoma, or uncontrolled central nervous system metastases.

### Statistical considerations and analyses

The RP2D was defined as the highest dose level in which at most one of six patients experienced a dose‐limiting toxicity. The primary objective of phase 2 was to evaluate preliminary evidence of antitumor activity by testing the null hypothesis that the ORR is less than 35% versus the alternative that it is greater, which was based on the historical ORR in patients treated with pembrolizumab monotherapy.^34^ The trial was conducted as an optimal Simon two‐stage design having 80% power to detect an ORR of 55% with one‐sided statistical testing performed at the 5% level of significance. The design called for 14 patients to be enrolled in the first stage and the study would be terminated if five or fewer responded. Otherwise, if five or more patients responded, an additional 30 patients were to be enrolled in the second stage. If 21 or more of the total 44 patients responded, the treatment might be deemed worthy of further investigation.

The incidence of treatment‐emergent adverse events was summarized by type of adverse event, grade and attribution with the most severe grade per patient being reported. ORR was defined as the proportion of patients with a confirmed CR or PR. The DCR was defined as the proportion of patients with a confirmed CR, PR, or stable disease (SD). The initial disease evaluation after treatment initiation was approximately 3 months, and there was no protocol‐defined minimum duration of SD. PFS was defined as the time from study treatment initiation to the date of first documentation of disease progression or death due to any cause. Otherwise, patients were censored at date of last radiographic assessment. OS was defined as the time from study treatment initiation to death due to any cause. Patients still alive were censored at the last date known to be alive. Survival probabilities were estimated and plotted using the Kaplan–Meier method. Estimates along with 95% pointwise confidence intervals are reported.

## RESULTS

A total of 28 participants started the study treatment between September 2019 and March 2024 and were included in the reported safety analysis. In phase 1b, eight patients were treated at cabozantinib doses of 40 mg (*n* = 6) and 60 mg (*n* = 2) in combination with pembrolizumab. In phase 2, a total of 20 patients were enrolled and treated at the RP2D of cabozantinib 40 mg in combination with pembrolizumab and were included in the efficacy analysis. Fourteen patients were accrued in the first stage of the two‐stage design. Six of those patients responded and accrual to the second stage started. However, the study was terminated after a total of 20 patients were accrued in phase 2 due to low accrual in the setting of evolving standard or care.

### Patient characteristics

In phase 1b, eight patients with a median age of 59 years (range, 49–79) were enrolled (Table 1). Six (75%) were female and two (25%) were male. Three (43%) were *BRAF* mutation‐positive, four (57%) were *BRAF* mutation‐negative, and one with missing information. None (0%) of the patients had prior chemotherapy, five (63%) had history of prior surgery, and one (12.5%) had prior adjuvant anti–PD‐1 immunotherapy. Baseline median lactate dehydrogenase (LDH) was 209 (range, 174–231). All patients were of non‐Hispanic White ethnicity. Sites of metastatic spread at time of trial enrollment included lymph nodes (*n* = 5), lung (*n* = 3), bone (*n* = 2), orbit (*n* = 1), liver (*n* = 1), stomach (*n* = 1), duodenum (*n* = 1), and colon (*n* = 1).

**TABLE 1:** Patient demographics and baseline characteristics.

Covariate	Statistics	Level	Phase 1 *N* = 8	Phase 2 *N* = 20
Sex	*N* (Col %)	Female	6 (75.0)	6 (30.0)
	*N* (Col %)	Male	2 (25.0)	14 (70.0)
Race	*N* (Col %)	White	8 (100)	20 (100)
Ethnicity	*N* (Col %)	Non‐Hispanic	8 (100)	20 (100)
Prior surgery	*N* (Col %)	No	3 (37.5)	6 (30.0)
	*N* (Col %)	Yes	5 (62.5)	14 (70.0)
Prior other treatment	*N* (Col %)	No	7 (87.5)	16 (80.0)
	*N* (Col %)	Yes	1 (12.5)	4 (20.0)
ECOG	*N* (Col %)	0	7 (87.5)	14 (75.0)
	*N* (Col %)	1	1 (12.5)	5 (25.0)
BRAF	*N* (Col %)	Positive	3 (42.9)	8 (42.1)
	*N* (Col %)	Negative	4 (57.1)	10 (52.6)
	*N* (Col %)	Indeterminant	0 (0)	1 (5.3)
	*N* (Col %)	Missing	1	1
Age, years	Median		59	55
	(Min–Max)		(49–79)	(27–80)
Baseline LDH	Median		209	218
(ref: 135–225 U/L)	(Min–Max)		(174–231)	(147–1391)

Abbreviations: Col %, column percentage; ECOG, Eastern Cooperative Oncology Group; LDH, lactate dehydrogenase; Max, maximum; Min, minimum.

In phase 2, an additional 20 patients with a median age of 55 years (range, 27–80) were enrolled (Table 1). Six (30%) were female and 14 (70%) were male. Eight (42%) were *BRAF* mutation‐positive, 10 (53%) were *BRAF* mutation‐negative, one (5%) was indeterminant, and one with missing information. Fourteen (70%) had prior surgical resection of their primary melanoma, and three (15%) had prior adjuvant anti–PD‐1 immunotherapy, with last adjuvant treatments received at least 10 months before trial enrollment. One patient had received prior BRAF/MEK‐directed therapy. Baseline median LDH was 218 (range, 147–1391). All patients were of non‐Hispanic White ethnicity. Sites of metastatic spread at time of trial enrollment included lymph nodes (*n* = 15), lung (*n* = 4), parotid gland (*n* = 2), subcutaneous soft tissues (*n* = 1), adrenal gland (*n* = 1), liver (*n* = 1), bone (*n* = 1), and colon (*n* = 1).

The full list of primary disease site, metastatic disease site, and LDH at study enrollment can be found in Table S1. None of the patients in this study had documented acral lentiginous melanoma.

### Safety

All 28 participants experienced at least one treatment‐related adverse event (AE), and serious AEs occurred in eight (27%) patients, of which six were treatment‐related. The most common treatment‐related AEs seen with the combination therapy were ALT elevation (71%; in two patients treated with 60 mg and 18 patients treated with 40 mg of cabozantinib), AST elevation (64%), hypophosphatemia (64%), fatigue (61%), alkaline phosphatase elevation (54%), anorexia (54%), diarrhea (54%), hypertension (43%), and nausea (43%) (Table 2). The most common grade ≥3 toxicities were hypertension (36%), hypokalemia (18%), hypophosphatemia (14%), and ALT elevation (14%). The frequency of all reported treatment‐emergent AEs is listed in Table S2.

**TABLE 2:** Adverse events possibly, probably or definitely related to study treatment occurring in ≥10% of patients in the safety population (*n* = 28).

	Grade, No. (%)				
Toxicity	1	2	3	4	Total, No. (%)
Any toxicity		6 (21)	18 (64)	4 (14)	28 (100)
Alanine aminotransferase increased	7 (25)	9 (32)	3 (11)	1 (4)	20 (71)
Aspartate aminotransferase increased	11 (39)	5 (18)	1 (4)	1 (4)	18 (64)
Hypophosphatemia	5 (18)	9 (32)	4 (14)		18 (64)
Fatigue	11 (39)	6 (21)			17 (61)
Alkaline phosphatase increased	12 (43)	2 (7)	1 (4)		15 (54)
Anorexia	13 (46)	2 (7)			15 (54)
Diarrhea	7 (25)	8 (29)			15 (54)
Hypertension		2 (7)	9 (32)	1 (4)	12 (43)
Nausea	11 (39)	1 (4)			12 (43)
Hypokalemia	5 (18)	1 (4)	5 (18)		11 (39)
Hypoalbuminemia	6 (21)	3 (11)			9 (32)
Proteinuria	6 (21)	3 (11)			9 (32)
Weight loss	3 (11)	6 (21)			9 (32)
Headache	8 (29)				8 (29)
Hypothyroidism	5 (18)	3 (11)			8 (29)
Oral pain	5 (18)	3 (11)			8 (29)
Rash maculopapular	4 (14)	3 (11)	1 (4)		8 (29)
Urine discoloration	8 (29)				8 (29)
Dysgeusia	3 (11)	4 (14)			7 (25)
Hypocalcemia	5 (18)	2 (7)			7 (25)
GGT increased	2 (7)	2 (7)	2 (7)		6 (21)
Hyponatremia	6 (21)				6 (21)
Lymphocyte count decreased	2 (7)	2 (7)	2 (7)		6 (21)
Neutrophil count decreased	3 (11)	3 (11)			6 (21)
Vomiting	5 (18)	1 (4)			6 (21)
Anemia	5 (18)				5 (18)
Blood bilirubin increased	4 (14)		1 (4)		5 (18)
Dry mouth	5 (18)				5 (18)
Hematuria	5 (18)				5 (18)
Mucositis oral	3 (11)	1 (4)	1 (4)		5 (18)
Palmar–plantar erythrodysesthesia syndrome	3 (11)	2 (7)			5 (18)
White blood cell decreased	4 (14)	1 (4)			5 (18)
Abdominal pain	2 (7)	1 (4)	1 (4)		4 (14)
Arthralgia	3 (11)		1 (4)		4 (14)
Dry skin	4 (14)				4 (14)
Dyspepsia	3 (11)	1 (4)			4 (14)
Generalized muscle weakness	4 (14)				4 (14)
Skin and subcutaneous tissue disorders—other, specify	3 (11)	1 (4)			4 (14)
Creatinine increased	3 (11)				3 (11)
Hyperkalemia	2 (7)		1 (4)		3 (11)
Platelet count decreased	3 (11)				3 (11)

Abbreviation: GGT, gamma‐glutamyl transferase.

### Determination of RP2D

In phase 1b, an initial cohort of three patients was treated with 40 mg of cabozantinib, and one patient experienced a dose limiting toxicity (DLT). A second cohort of three patients was treated with 40 mg of cabozantinib. In total, one of six patients experienced a DLT, and the dose was escalated to 60 mg of cabozantinib. Two patients were treated with 60 mg of cabozantinib. Although neither patient experienced a DLT, both developed elevated liver function tests requiring study treatment to be stopped permanently (after two cycles of treatment, one patient developed grade 3 increased ALT, another patient developed grade 4 increased ALT and AST). The RP2D of cabozantinib 40 mg dose was carried forward to phase 2.

### Efficacy

**FIGURE 1:**
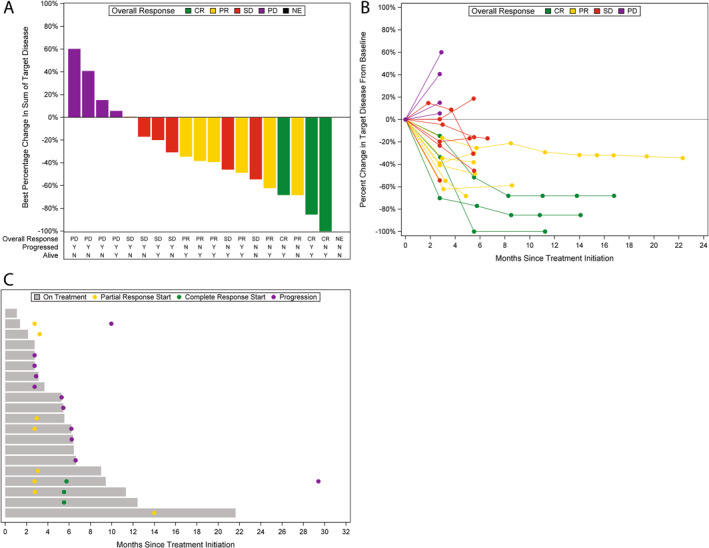
Change in target lesions from baseline. (A) Best percentage change in the sum of target disease from baseline. (B) Percentage change in the sum of target disease from baseline. (C) Treatment duration and disease status over time. CR indicates complete response; NE, not evaluated; PD, progression of disease; PR, partial response; SD, stable disease.

**FIGURE 2:**
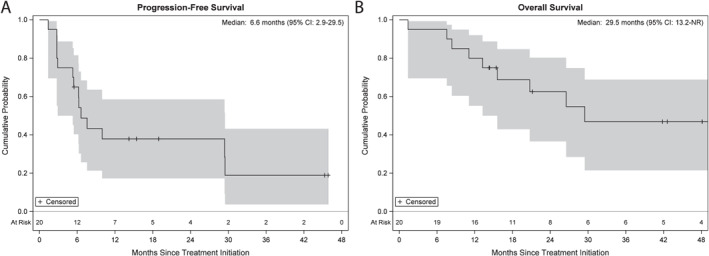
Progression‐free survival (A) and overall survival (B) Kaplan–Meier curves and 95% pointwise confidence intervals.

The median duration of treatment was 5.5 months (range, 1.1–21.6). Two (10%) patients completed treatment per study protocol criteria, whereas 18 (90%) patients did not complete treatment due to disease progression (*n* = 9), side effects (*n* = 6), treating physician’s discretion (*n* = 2), and patient withdrawal/refusal (*n* = 1). The DCR was 75% (95% CI, 51%–91%) and the ORR was 45% (95% CI, 23%–68%). Three (15%) patients achieved CR, six (30%) patients achieved PR, six (30%) patients had SD, four (20%) patients had PD, and one (5%) patient was not evaluable (Figure 1A). The change in the sum of target disease in relation to other efficacy end points and over time is shown in Figure 1B. Patient’s treatment duration and disease status over time are depicted in Figure 1C. At a median follow‐up of 20.9 months (range, 1.4–53.8), 11 patients had progressed, and nine patients had died. The median PFS (mPFS) was 6.6 months (95% CI, 2.9–29.5) (Figure 2A). The median OS (mOS) was 29.5 months (95% CI, 13.2–not reached) (Figure 2B).

## DISCUSSION

Here, we present the results of a phase 1b/2 clinical trial of cabozantinib, a multitarget TKI with specificity for MET, VEGFR2, RET, and AXL, in combination with pembrolizumab in patients with previously untreated metastatic or recurrent unresectable cutaneous melanoma.^29^ Cabozantinib 40 mg was selected as the RP2D based on data from the phase 1b portion of the study, with one of six patients experiencing a DLT at this dose. In the phase 2 portion of this study, only two (10%) of patients completed treatment per study protocol, with the nine (45%) withdrawing due to disease progression, six (30%) due to side effects, and two (10%) due to treating physician discretion. Furthermore, all 28 patients enrolled in this trial were non‐Hispanic White, which limits generalizability of this study. Main grade ≥3 TRAEs included hypertension (36%), hypokalemia (18%), hypophosphatemia (14%), and ALT elevation (14%). Per pre‐specified statistical criteria in a Simon two‐stage design, after observing treatment responses in six of 14 initially enrolled patients, the second stage of the phase 2 was opened with planned enrollment of 30 more patients for planned total enrollment of 44 patients. The phase 2 portion of the study was terminated by the investigators after enrollment of 20 patients due to low accrual in the setting of evolving standard of care, as described in greater detail below. Efficacy measures from available data show ORR 45% (95% CI, 23%–68%), numerically comparing favorably to prior data on single agent anti–PD‐1 therapy, although wide confidence intervals prevent drawing strong conclusions. Median PFS was 6.6 (95% CI, 2.9–29.5) months and median OS was 29.5 (95% CI, 13.2–not estimable) months.

At the time of initiation of this trial in 2019, options for frontline therapy for advanced melanoma included monotherapy with pembrolizumab or nivolumab, or dual ICI therapy with ipilimumab and nivolumab, although with 55%–60% risk of grade ≥3 TRAEs, and 40% of patients interrupting treatment due to toxicity.^35^ The purpose of this trial was to explore whether a TKI + anti–PD‐1 combination could enhance efficacy relative to anti–PD‐1 monotherapy, estimated to be 31%–33%.^9^, ^10^ The selection of cabozantinib in combination with pembrolizumab in this study was based on phase 2 data showing single‐agent efficacy of cabozantinib, as well as a substantial body of preclinical and clinical evidence supporting TKI‐mediated potentiation of anti‐PD1 activities^20^ and immunogenic cell death,^21^ contributing to durable systemic antitumor immunity, as has been observed in other solid tumors such as RCC ^22^, ^24^, ^25^, ^26^, ^27^ and endometrial cancer.^28^ We reasoned that such an ICI and TKI combination would meet a clinical need for patients with advanced melanoma who are unfit for dual ICI therapy.

Since this trial began in 2019, frontline management of advanced or metastatic melanoma has evolved substantially. The CheckMate 511 trial subsequently defined alternative dosing of ipilimumab 1 mg/kg with nivolumab 3 mg/kg as having similar antitumor efficacy, but significantly better tolerability compared to prior standard dosing of ipilimumab 3 mg/kg and nivolumab 1 mg/kg defined in the CheckMate 067 trial, with risk of grade ≥3 TRAEs of 34% versus 48%, descriptive *p* value = .006.^32^, ^36^ In addition, a second very tolerable dual ICI combination of nivolumab with relatimab was shown to yield improved mPFS (10.1 vs. 4.6 months) compared to single agent nivolumab in the interim analysis of the phase 2/3 RELATIVITY‐047 trial.^14^ By contrast, a phase 2 study of nivolumab/ipilimumab plus cabozantinib in advanced melanoma (NCT04091750) that started accrual in 2020 was terminated early in 2023 based on its interim analysis demonstrating that the study did not meet the predefined PFS threshold.^37^

Another TKI lenvatinib was combined with pembrolizumab in phase 2 LEAP‐004 study in metastatic melanoma patients with confirmed progression on anti–PD‐1 ± anti–CTLA‐4 therapy. Among the 103 participants, the ORR was 21.4% (95% CI, 13.9–30.5).^38^ Notably, a higher ORR of 33% was observed in the subgroup with documented progression after dual anti–PD‐1 and anti–CTLA‐4 blockade. Building on these findings, the LEAP‐003 trial investigated lenvatinib plus pembrolizumab as first‐line therapy for unresectable or metastatic melanoma. A total of 674 patients were randomized to receive either the combination regimen or pembrolizumab with placebo. Although the initial interim analysis suggested an improvement in median PFS in the lenvatinib arm, this advantage was not sustained in subsequent analyses, ultimately leading to early termination of the study.^39^ The negative outcomes of LEAP‐003, combined with evolving standards of care for first‐line treatment, prompted investigators to discontinue the trial prematurely.

Despite these findings, it remains reasonable to propose that cabozantinib, given its distinct kinase inhibition profile compared with lenvatinib, may warrant further investigation. We believe that the safety and antitumor activity data generated in the present study will provide a valuable foundation for the design of future clinical trials exploring novel TKI and immune checkpoint inhibitor combination strategies.

## AUTHOR CONTRIBUTIONS

**Yousef Zakharia**: Conceptualization; data curation; writing—review and editing. **Donghyun Kim**: Validation; writing—original draft; writing—review and editing. **Michele Freesmeier**: Data curation; writing—review and editing. **Melanie Frees**: Data curation; writing—review and editing. **Sarah Mott**: Formal analysis; validation; writing—original draft; writing—review and editing. **Varun Monga**: Data curation; writing—review and editing. **Douglas Laux**: Data curation; writing—review and editing. **Asad Javed**: Data curation; writing—review and editing. **John Rieth**: Data curation; writing—original draft; writing—review and editing. **John Smestad**: Writing—review and editing. **Mohammed Milhem**: Conceptualization; data curation; writing—review and editing.

## CONFLICT OF INTEREST STATEMENT

Varun Monga reports consulting fees from Atheneum Consulting and ClearView Healthcare Partners; fees for other professional activities from Association of Northern California Oncologists, Binaytara Foundation, Iowa Oncology Society, Nested Therapeutics, Polaris, Prelude Therapeutics, Replimune, and Vivace Therapeutics; and grant and/or contract funding from InhibRx and Replimune. John Rieth reports fees for professional activities from Deciphera Pharmaceuticals and EMD Serono. Yousef Zakharia serves on the advisory boards of Bristol‐Myers Squibb, EMD Serono, Exelixis, Gilead, Janssen, Pfizer, Arcus Biosciences, and Seagen. The other authors declare no conflicts of interest.

## Supporting information

Table S1

Table S2

## Data Availability

The data that support the findings of this study are available on request from the corresponding author. The data are not publicly available due to privacy or ethical restrictions.
