# Supplementary material for: A phase 1b/2 study of cabozantinib in combination with pembrolizumab in advanced cutaneous melanoma
Source: Cancer. 2026 Feb 17;132(4):e70326. doi: 10.1002/cncr.70326 (PMC12912253; doi:10.1002/cncr.70326)
Supplement: Supplementary file 1 — Table S1 [file CNCR-132-e70326-s002.docx]

**Supplemental Table 1. Primary site, metastatic site and LDH at study enrollment**

| **Patient**  **ID** | **Study**  **Phase** | **Primary**  **Site** | **Metastatic**  **Site** | **LDH**  **(ref: 135-225 U/L)** |
| --- | --- | --- | --- | --- |
| 1 | Ib | Left back | Left axillary lymph node | 231 (H) |
| 2 | Ib | Scalp | Lung | 229 (H) |
| 3  4  5  6  7  8  9  10  11  12  13  14  15  16  17  18  19  20  21  22  23  24  25  26  27  28 | Ib | Right shoulder | Right orbit, bone, liver | 200 |
|  | Ib  Ib  Ib  Ib  Ib  II  II  II  II  II II II II II II  II  II II II  II  II  II  II  II  II | Right temple  Unknown  Unknown  Unknown  Unknown  Left leg  Right back  Unknown  Left neck  Left chin  Right back  Back  Right temple  Left temple  Left leg  Unknown  Right arm  Unknown  Left arm  Scalp  Unknown  Right leg  Back  Left shoulder  Left shoulder | Right axillary lymph node, lung  Duodenum  Right inguinal lymph node  Right axillary lymph node  Mediastinal lymph node, bone, lung, stomach, colon  Left inguinal lymph node  Bilateral inguinal lymph nodes  Mesenteric lymph nodes, left adrenal gland, lung  Left cervical lymph node, left parotid gland  Lung  Right axillary lymph node  Bones, liver, lung  Right parotid gland  Lung  Left iliac lymph node  Right iliac lymph node  Right axillary lymph node  Left axillary lymph node  Left axillary lymph node  Left preauricular lymph node  Retroperitoneal lymph node  Left inguinal lymph node, cecum  Subcutaneous nodules  Left axillary lymph node  Left axillary lymph node | 209  214  202  221  174  217  400 (H)  494 (H)  189  169  215  1391 (H)  196  432 (H)  241 (H)  209  192  524 (H)  232 (H)  199  297 (H)  247 (H)  147  193  220 |

(H): higher than reference range
