# Supplementary material for: A phase 1b/2 study of cabozantinib in combination with pembrolizumab in advanced cutaneous melanoma
Source: Cancer. 2026 Feb 17;132(4):e70326. doi: 10.1002/cncr.70326 (PMC12912253; doi:10.1002/cncr.70326)
Supplement: Supplementary file 2 — Table S2 [file CNCR-132-e70326-s001.docx]

**Supplemental Table 2. Treatment-emergent adverse events occurring in ≥10% of patients in the safety population (n=28).**

|  | **Grade** | | | |  |
| --- | --- | --- | --- | --- | --- |
| **Toxicity** | **1-2** | **3** | **4** | **5** | **Total** |
| Any Toxicity | 5 (18%) | 18 (64%) | 4 (14%) | 1 (4%)^†^ | 28 (100%) |
| Hypophosphatemia | 17 (61%) | 4 (14%) |  |  | 21 (75%) |
| Alanine aminotransferase increased | 16 (57%) | 3 (11%) | 1 (4%) |  | 20 (71%) |
| Aspartate aminotransferase increased | 17 (61%) | 1 (4%) | 1 (4%) |  | 19 (68%) |
| Fatigue | 19 (68%) |  |  |  | 19 (68%) |
| Alkaline phosphatase increased | 14 (50%) | 1 (4%) |  |  | 15 (54%) |
| Anorexia | 15 (54%) |  |  |  | 15 (54%) |
| Diarrhea | 15 (54%) |  |  |  | 15 (54%) |
| Nausea | 13 (46%) |  |  |  | 13 (46%) |
| Urine discoloration | 13 (46%) |  |  |  | 13 (46%) |
| Hypertension | 2 (7%) | 9 (32%) | 1 (4%) |  | 12 (43%) |
| Hypoalbuminemia | 11 (39%) | 1 (4%) |  |  | 12 (43%) |
| Hypokalemia | 7 (25%) | 5 (18%) |  |  | 12 (43%) |
| Proteinuria | 11 (39%) |  |  |  | 11 (39%) |
| Anemia | 8 (29%) | 1 (4%) |  |  | 9 (32%) |
| Hypocalcemia | 8 (29%) |  | 1 (4%) |  | 9 (32%) |
| Rash maculo-papular | 8 (29%) | 1 (4%) |  |  | 9 (32%) |
| Weight loss | 9 (32%) |  |  |  | 9 (32%) |
| Abdominal pain | 7 (25%) | 1 (4%) |  |  | 8 (29%) |
| Dysgeusia | 8 (29%) |  |  |  | 8 (29%) |
| GGT increased | 6 (21%) | 2 (7%) |  |  | 8 (29%) |
| Headache | 8 (29%) |  |  |  | 8 (29%) |
| Hyponatremia | 6 (21%) | 2 (7%) |  |  | 8 (29%) |
| Hypothyroidism | 8 (29%) |  |  |  | 8 (29%) |
| Lymphocyte count decreased | 5 (18%) | 3 (11%) |  |  | 8 (29%) |
| Oral pain | 8 (29%) |  |  |  | 8 (29%) |
| Vomiting | 8 (29%) |  |  |  | 8 (29%) |
| Hematuria | 7 (25%) |  |  |  | 7 (25%) |
| Blood bilirubin increased | 5 (18%) | 1 (4%) |  |  | 6 (21%) |
| Neutrophil count decreased | 6 (21%) |  |  |  | 6 (21%) |
| Pain | 5 (18%) | 1 (4%) |  |  | 6 (21%) |
| Arthralgia | 4 (14%) | 1 (4%) |  |  | 5 (18%) |
| Cough | 5 (18%) |  |  |  | 5 (18%) |
| Dry mouth | 5 (18%) |  |  |  | 5 (18%) |
| Dyspnea | 5 (18%) |  |  |  | 5 (18%) |
| Mucositis oral | 4 (14%) | 1 (4%) |  |  | 5 (18%) |
| Palmar-plantar erythrodysesthesia syndrome | 5 (18%) |  |  |  | 5 (18%) |
| Skin and subcutaneous tissue disorders - Other, specify | 5 (18%) |  |  |  | 5 (18%) |
| White blood cell decreased | 5 (18%) |  |  |  | 5 (18%) |
| Creatinine increased | 4 (14%) |  |  |  | 4 (14%) |
| Dry skin | 4 (14%) |  |  |  | 4 (14%) |
| Dyspepsia | 4 (14%) |  |  |  | 4 (14%) |
| General disorders and administration site conditions - Other, specify | 3 (11%) | 1 (4%) |  |  | 4 (14%) |
| Generalized muscle weakness | 4 (14%) |  |  |  | 4 (14%) |
| Hyperkalemia | 3 (11%) | 1 (4%) |  |  | 4 (14%) |
| Hypomagnesemia | 4 (14%) |  |  |  | 4 (14%) |
| Platelet count decreased | 4 (14%) |  |  |  | 4 (14%) |
| Sore throat | 4 (14%) |  |  |  | 4 (14%) |
| Constipation | 3 (11%) |  |  |  | 3 (11%) |
| Dizziness | 3 (11%) |  |  |  | 3 (11%) |
| Fever | 3 (11%) |  |  |  | 3 (11%) |
| Musculoskeletal and connective tissue disorder - Other, specify | 3 (11%) |  |  |  | 3 (11%) |
| Myalgia | 3 (11%) |  |  |  | 3 (11%) |

† This Grade 5 event was death due to disease progression which was documented as an adverse event per study protocol.
